# Ratiometric BRET Measurements of ATP with a Genetically-Encoded Luminescent Sensor

**DOI:** 10.3390/s19163502

**Published:** 2019-08-10

**Authors:** Se-Hong Min, Alexander R. French, Keelan J. Trull, Kiet Tat, S. Ashley Varney, Mathew Tantama

**Affiliations:** 1 Department of Chemistry, Purdue University, 560 Oval Drive, West Lafayette, IN 47907, USA; 2 Hall for Discovery Learning 399, Purdue Institute for Integrative Neuroscience, 207 South Martin Jischke Drive, West Lafayette, IN 47907, USA; 3 Department of Chemistry, Wellesley College, 106 Central Street, Wellesley, MA 02481, USA

**Keywords:** bioluminescence, BRET, ATP, genetically-encoded biosensor, NanoLuc, mScarlet

## Abstract

Luciferase-based reporters provide a key measurement approach in a broad range of applications, from in vitro high-throughput screening to whole animal imaging. For example, luminescence intensity is widely used to measure promoter activity, protein expression levels, and cell growth. However, luminescence intensity measurements are subject to quantitative irregularities caused by luminescence decay and variation in reporter expression level. In contrast, bioluminescence resonance energy transfer (BRET) sensors provide the advantages of luciferase-based reporters but overcome the aforementioned irregularities because of the inherently ratiometric readout. Here, we generated a new ratiometric BRET sensor of ATP (ARSeNL—ATP detection with a Ratiometric mScarlet-NanoLuc sensor), and we demonstrated that it provides a stable and robust readout across protein, cell, and whole animal tissue contexts. The ARSeNL sensor was engineered by screening a color palette of sensors utilizing variants of the high photon flux NanoLuc luciferase as donors and a panel of red fluorescent proteins as acceptors. We found that the novel combination of NanoLuc and mScarlet exhibited the largest dynamic range, with a 5-fold change in the BRET ratio upon saturation with ATP. Importantly, the NanoLuc-mScarlet BRET pair provided a large spectral separation between luminescence emission channels that is compatible with green and red filter sets extensively used in typical biological microscopes and animal imaging systems. Using this new sensor, we showed that the BRET ratio was independent of luminescence intensity decay and sensor expression level, and the BRET ratio faithfully reported differences in live-cell energy metabolism whether in culture or within mouse tissue. In particular, BRET analyte sensors have not been used broadly in tissue contexts, and thus, in principle, our sensor could provide a new tool for in vivo imaging of metabolic status.

## 1. Introduction

Bioluminescence imaging has been widely adopted in biomedical research because it offers a cost-effective, easily accessible method for low background and high signal-to-noise non-invasive optical measurements [1,2,3,4,5]. For example, bioluminescent reporters have been extensively used with rodent cancer models to measure signaling, tumor volume, and immune cell infiltration in vivo [6,7]. Additionally, a number of luminescent probes have been developed for metals and ions [8,9,10,11,12], second messengers [13,14], enzyme activity [15,16,17], protein-protein interactions [18,19,20,21], and reactive species [22,23,24,25], but these are predominantly used for in vitro assays, cultured cell models, or instrument development. One challenge in the development of genetically-encoded luciferase reporters is that the rapid decay of luminescence intensity over time poses a substantial problem for stable signal measurement. Furthermore, the absolute luminescence intensity varies depending on the expression levels of genetically-encoded luciferase reporters. Thus, luminescence intensity is not an ideal readout for quantitative comparisons between independent specimens, such as cell assay treatment groups and animal models.

In contrast to intensity-based reporters, ratiometric sensors that employ intramolecular bioluminescence resonance energy transfer (BRET) provide an optical signal normalized for variations in luminescence flux caused by differences in sensor expression level or changing substrate concentration. However, ratiometric BRET sensors have not been widely used beyond in vitro applications, with notable exceptions, such as luminescent calcium imaging in brain slices and the detection of protein-protein interactions in vivo [12,18].

In this study, our objective was to demonstrate that a ratiometric BRET sensor provides an effective tool for live-cell measurements across model systems, from single-cell microscopy of cultured cells to macroscopic imaging through animal tissue. As our test case, we engineered a bioluminescent ATP sensor because of the broad importance of bioenergetic status as a fundamental metric of physiology. Furthermore, we showed that it is possible to use a red fluorescent protein (RFP) acceptor to create a BRET sensor with a large donor-acceptor emission wavelength difference that facilitates filter-based imaging in widely available animal imaging systems.

## 2. Materials and Methods

### 2.1. Materials

Unless otherwise noted, chemicals were purchased from Sigma (St. Louis, MO, USA), enzymes from New England Biolabs (Ipswich, MA, USA), and cell culture reagents from ThermoFisher (Waltham, MA, USA). Coelenterazine-h was purchased from Biotium (Fremont, CA, USA) and Nano-Glo furimazine reagent from Promega (Madison, WI, USA).

### 2.2. Protein Engineering and Library Screen

Sensors were constructed by Gibson Assembly using the NEB HiFi kit, sub-cloned into the pRSETB vector for expression as His-tagged protein in BL21(DE3) *Escherichia coli*, and purified by nickel-affinity chromatography. CeNL/pcDNA3 was a gift from Takeharu Nagai (Addgene plasmid #85199), pcDNA3-mNeonGreen was a gift from Richard Day (Indiana University), pCytERm-mScarlet_N1 was a gift from Dorus Gadella (Addgene plasmid #85066), and pBad-HisB-RRvT (Addgene plasmid #87364) and pBad-HisB-GRvT (Addgene plasmid #87363) were gifts from Robert Campbell. The fluorescence and luminescence ATP dose-response curves for purified protein solutions were measured on a BioTek (Winooski, VT, USA) Synergy H4 multi-mode microplate reader at ambient temperature. Luminescence was also measured using a Spectral Instruments Ami HT (Tuscon, AZ, USA). Plasmids generated in this study are distributed via Addgene (Watertown, MA, USA).

### 2.3. Live Single-Cell Microscopy

The sensor genes were sub-cloned into the GW1 mammalian expression vector, and HEK293A cells were transfected by the calcium phosphate method [26]. Cells were imaged with 30 s exposure times and 2 min imaging interval on an Olympus IX83 microscope (Chelmsford, MA, USA) with a 40X 1.35 NA Apo oil objective, an Andor Zyla 4.2 sCMOS camera (South Windsor, CT, USA) at 2-by-2 pixel binning, and 470/24 nm and 632/60 nm emission filters. Just before the addition of furimazine, well volume was exchanged twice with imaging media without glucose. After adding furimazine, a three-point baseline was acquired. Subsequently, 10 mM 2-deoxyglucose (2DG) plus 2.5 μM oligomycin A was added for metabolic inhibition or 10 mM glucose plus DMSO was added for vehicle controls. Cells were imaged for ~30 min until luminescence decayed to the background. Image backgrounds were subtracted before calculating the ratio images of the luminescence channels using NIH ImageJ version 1.52.

### 2.4. Animal Imaging System

All animal procedures were performed in strict accordance with recommendations provided in the National Institutes of Health (NIH) Guide for the Care and Use of Laboratory Animals, according to protocols approved by the Purdue Animal Care and Use Committee and the Purdue University Laboratory Animal Program to minimize pain and suffering. Male and female adult Balb/c and FVB mice were sacrificed and then injected with protein solution or live cells subcutaneously in the hind limb area. NanoLuc substrate, 20 μM coelenterazine-h or 0.5X Promega Nano-Glo furimazine solution, was mixed with the sample just before injection. Protein, cells, and animals were imaged at ambient temperature using a Spectral Instruments Ami HT (SI Ami HT) equipped with a 510/20 nm and 650/20 nm emission filters.

### 2.5. Data Analysis

All measurements are shown as the mean ± standard deviation (sd). Unpaired, two-tailed Student’s *t*-test was used for comparison between the means of two groups.

## 3. Results

### 3.1. Protein Engineering and Library Screen

**Figure 1:**
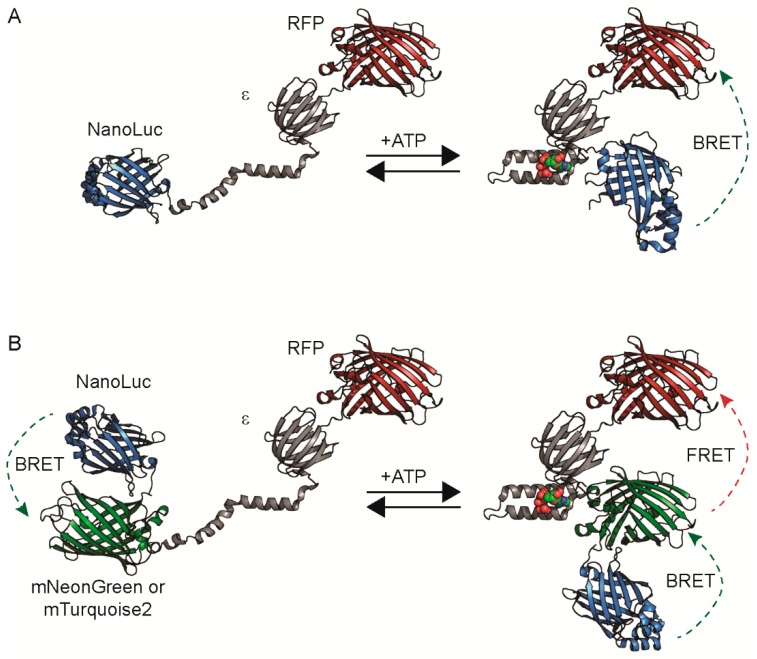
Sensor designs. Diagrams of a bioluminescence resonance energy transfer (BRET)-only sensor (**A**) or BRET-FRET (Förster-type resonance energy transfer) sensor (**B**) depict NanoLuc in blue, the *B. subtilis* ε subunit in grey, an RFP (red fluorescent protein) in red, and either mNeonGreen or mTurquoise2 in green.

We first re-engineered the fluorescent Förster-type resonance energy transfer (FRET)-based ATeam1.03 ATP sensor into a color palette of bioluminescence resonance energy transfer (BRET) sensors. The ATeam1.03 sensor consists of a cyan fluorescent protein (CFP) and yellow fluorescent protein (YFP) FRET pair flanking the ATP-binding ε subunit of the ATP synthase from *Bacillus subtilis* [27]. When ATP binds to the ε subunit, a large conformational change brings the fluorescent proteins into close proximity, increasing FRET. For our library construction, we replaced the CFP-YFP pair with a bioluminescent donor and fluorescent protein acceptor (Figure 1).

For the acceptor fluorescent protein, we tested tdTomato [28], RRvT [29], GRvT [29], and mScarlet [30] because these RFPs provide a large spectral distance between donor and acceptor luminescence peaks, facilitating filter-based imaging. We chose tdTomato because of its potential for efficient BRET [31] and its derivative, RRvT, because of its increased brightness. We also tested the long Stokes shift tdTomato-derivative, GRvT, given its large spectral overlap with NanoLuc. Lastly, we chose mScarlet because it is the brightest monomeric RFP to date, and it can serve as an efficient FRET acceptor to cyan-emitting donors in the same spectral range as NanoLuc emission [32].

We screened these RFP acceptors in combination with three different bioluminescent donors that we classify into two designs: BRET-only sensors and BRET-FRET dual-mode sensors (Figure 1 and Figure S1).

The BRET-only sensors use NanoLuc alone as the bioluminescent donor. We tested different orientations, with NanoLuc on either the N- or C-terminus. Interestingly, we found that constructs with NanoLuc on the N-terminus and the RFP acceptor on the C-terminus of the ε subunit typically led to poor or no expression in bacteria. Thus, in our second design, we only generated BRET-FRET sensors with the RFP on the N-terminus.

The BRET-FRET sensors utilize one of the fluorescent protein-NanoLuc fusions, CeNL (mTurquoise2-NanoLuc) or GeNL (mNeonGreen-NanoLuc), because they have higher brightness compared to NanoLuc alone [31]. These CeNL and GeNL-based sensors enable dual-modality imaging as BRET-FRET sensors because of the FRET between the RFP and the mTurquoise2 or mNeonGreen [9,33]. For comparison, we also generated the mNeonGreen-CeNL pairing because mNeonGreen and mTurquoise2 can act as a high-efficiency FRET pair [32,34,35].

In the initial screen of these sensors, we first measured the FRET and BRET dynamic ranges after the addition of saturating ATP (Table 1). Many of the CeNL and GeNL-utilizing BRET-FRET constructs exhibited dynamic ranges with a near 100% maximal change, similar to the original ATeam1.03 [27]. Furthermore, the ATP affinity was not affected by the changes in donor and acceptor, underscoring the robustness of the ε subunit’s ability to tolerate substitutions at its termini (Figure S2). Interestingly, the magnitude of the FRET dynamic range did not correlate with or predict the BRET dynamic range (Table 1), and the lower BRET dynamic ranges were not caused by higher resting BRET. For example, the mNeonGreen-CeNL pairing resulted in one of the highest FRET dynamic ranges but the lowest BRET dynamic range. Conversely, the RRvT-GeNL pairing exhibited a higher BRET dynamic range but a lower FRET dynamic range. It may be that direct interactions between the NanoLuc and RFP in the BRET mode affect the overall dynamic range in ways that cannot be predicted by the performance of the FRET pairs.

Importantly, we found that the BRET-only sensors exhibited excellent dynamic ranges of >400%, exceeding the already good performance of the BRET-FRET sensors (Table 1). These results are interesting, given that GeNL and CeNL are brighter than NanoLuc alone, and both spectrally red-shift the NanoLuc emission to better overlap with the RFP absorption [31]. It is possible that the lower BRET dynamic range in the GeNL and CeNL constructs was caused by the additional bulk of the mTurquoise2 or mNeonGreen, which might restrict the CeNL and GeNL in a conformation that is sub-optimal for BRET in the ATP-bound state. Similarly, the use of GRvT in the BRET-only constructs did not improve BRET with NanoLuc compared to tdTomato in the sensor or a control construct lacking the ε subunit (Figure S3) despite the greater overlap between NanoLuc emission and GRvT excitation spectra (Table 1).

**Figure 2:**
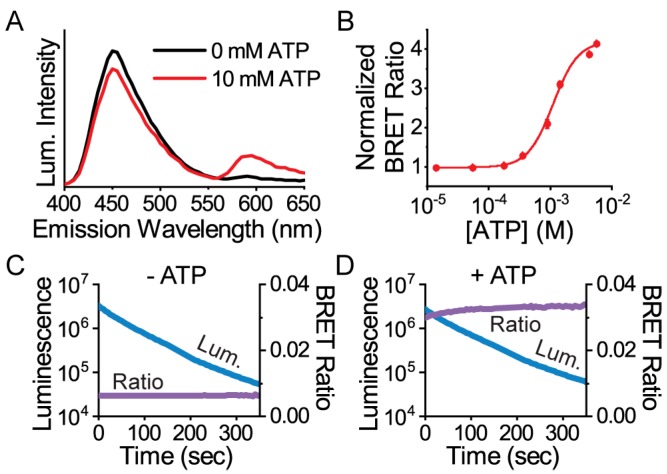
Protein characterization of the mScarlet-ε-NanoLuc sensor called ARSeNL (ATP detection with a Ratiometric mScarlet-NanoLuc sensor). (**A**) Luminescence emission spectra and (**B**) the ATP-dependent mScarlet/NanoLuc BRET ratio response (K_D_ = 1.1 ± 0.1 mM, *n* = 3) were measured on a microplate reader, and data were fitted to the Hill equation. (**C**,**D**) The BRET ratio (purple) is stable over time, even while the total luminescence intensity (blue) decays rapidly. The BRET ratio is (**C**) 0.0063 ± 0.0001 in the presence of 0 mM ATP and (**D**) 0.0324 ± 0.009 in the presence of 10 mM ATP (*n* = 3).

Instead, the mScarlet-NanoLuc pair worked best with a low resting BRET in the absence of ATP and a greater than a 4-fold maximal change in BRET ratio upon ATP saturation (Figure 2). Characterization of the protein in solution demonstrated that the sensor exhibited an apparent ATP affinity (K_D_ = 1.1 ± 0.1 mM, *n* = 3) in agreement with the original ATeam1.03 at ambient temperature. Importantly, whether in the ATP-free or ATP-bound state, the BRET ratio was stable over time even during a 10-fold change in the NanoLuc intensity due to its luminescence decay (Figure 2). The stability of the BRET ratio is a key illustration of how ratiometric measurements facilitate quantitation with reduced artifacts compared to single-channel intensity measurements that drift significantly over time.

We named the mScarlet-ε-NanoLuc sensor ARSeNL (ATP detection with a Ratiometric mScarlet-NanoLuc sensor) and next demonstrated its use in imaging applications.

### 3.2. Live Single-Cell BRET Microscopy

**Figure 3:**
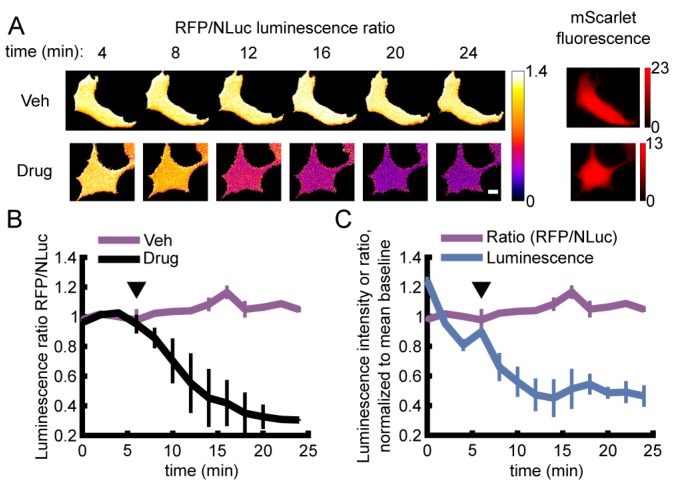
Live single-cell BRET microscopy. (**A**,**B**) HEK293A cells expressing ARSeNL were subjected to either metabolic inhibition with 10 mM 2DG (2-deoxyglucose) and 2.5 μM oligomycin A (Drug) or 10 mM glucose and DMSO (Veh) just before acquisition marked by the arrowhead. (**A**) BRET ratio images for typical cells undergoing each treatment at different time points. Scale bar: 10 μm. (**B**) The sensor BRET ratio faithfully reports the decrease in cellular ATP in inhibitor-treated cells (Drug, *n* = 4 independent wells, 7–9 cells/well) and no change in the glucose control (Veh, *n* = 3). Error bars represent mean ± sd. (**C**) The BRET ratio (purple) is stable over time, even as total luminescence intensity decays (blue). The BRET ratio (purple) is the same data shown in (**B**).

To test the ability of ARSeNL to report changes in cellular ATP, we expressed the sensor in HEK293A cells and carried out luminescence imaging on a standard widefield epifluorescence microscope. Metabolic inhibition was induced by the addition of the metabolic inhibitors 2DG and oligomycin A (Figure 3). At ambient temperature, metabolic inhibition caused a decrease in intracellular ATP levels within minutes that was reported as a 71% decrease in the absolute BRET ratio. This change is in good agreement with the 5-fold change from zero to saturating ATP in measurements with purified sensor (Table 1). In contrast, the BRET ratio did not decrease in glucose vehicle control (Figure 3).

Similar to the performance of the purified sensor protein (Figure 2), the BRET ratio of ARSeNL in vehicle-treated cells remained steady despite luminescence decay (Figure 3). Furthermore, integration times of only 30 s were sufficient to collect ample luminescence signal with an air-cooled sCMOS camera. Thus, the use of the bright NanoLuc luciferase simplifies imaging requirements compared to other luciferases that can require minutes of integration time, back-illuminated or electron-multiplying CCD cameras, or a combination of both [36,37,38,39]. Together, these results underscore the brightness and signal stability afforded by our ratiometric sensor design.

### 3.3. Macroscopic Imaging through Animal Tissue

**Figure 4:**
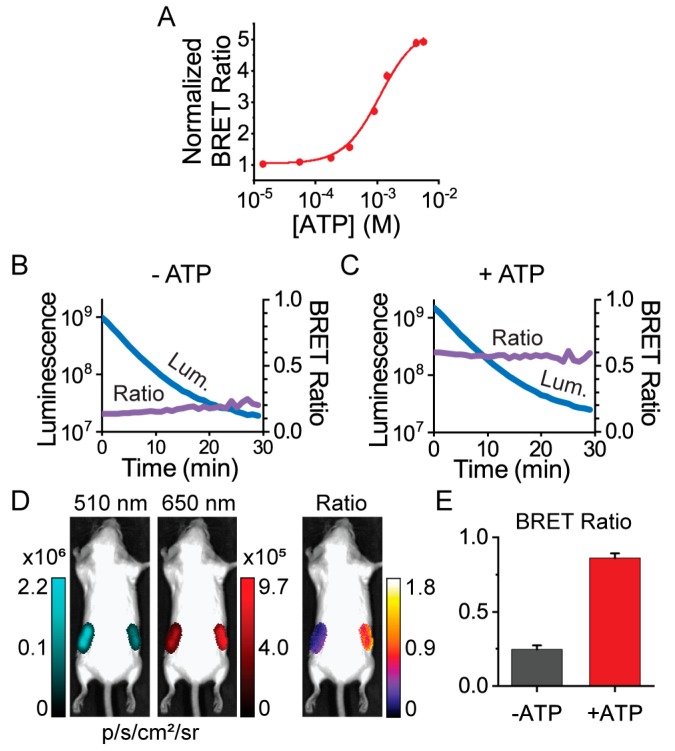
Ratiometric BRET imaging of ARSeNL purified protein using the Spectral Instruments (SI) Ami HT whole-animal imaging system. (**A**) The dynamic range is not affected by the use of standard GFP (510/20 nm) and RFP (650/20 nm) emission filters in the SI Ami HT imaging system (K_D_ = 1.1 ± 0.2 mM, *n* = 3). Data were fitted to the Hill equation. (**B**,**C**) The mScarlet/NanoLuc BRET ratio (purple) is stable over time in solutions containing 0 mM ATP (**B**) or 10 mM ATP (**C**) and 1 nM ARSeNL protein (*n* = 4), while the total luminescence intensity (blue) decays. (**D**,**E**) Whole-animal imaging of unshaved adult Balb/c mice. ARSeNL, pre-equilibrated with or without ATP, was placed subcutaneously in the left and right hindlimb areas, respectively, and the ATP-dependent ratio difference was preserved when imaged through tissue and fur (mean ± sd, *n* = 3, *p* < 0.001).

We next demonstrated that ARSeNL could serve as a ratiometric reporter of intracellular ATP when imaged through tissue. Our first objective was to characterize the spectral compatibility of the sensor with whole-animal imaging systems equipped with standard GFP and RFP emission filters that would be commonly accessible to individual labs or core facility users. In particular, the GFP filter collects emission that is off-peak for the NanoLuc donor luminescence, but given the high photon flux of NanoLuc, we hypothesized that our ratio signal would not be compromised despite instrumental losses. Indeed, we found that even with the imperfect emission filter matching in the SI Ami HT system, ARSeNL maintained a 5-fold dynamic range in its ATP dose-response using protein solutions (Figure 4). We also validated that the system could efficiently detect different levels of photon flux without causing artifacts in the BRET ratio, and in doing so we demonstrated that the BRET ratio faithfully reported differences in ATP levels in a protein concentration-independent manner (Figure S4). Furthermore, we found the BRET ratio was stable over time despite luminescence decay, and we were able to easily measure luminescence even at 1 nM protein concentrations (Figure 4). Thus, ARSeNL is bright enough that it can be effectively used for ratio imaging even without perfect wavelength-matching in the detection system.

Given these promising results, we next sought to establish whether the ATP-dependent ratio signal from ARSeNL is preserved when imaging through tissue in a whole animal. Xenograft cancer models in which tumor cells are injected and grow as a mass below the skin are one of the most common uses of bioluminescence imaging, and therefore we carried out ratiometric imaging of subcutaneous sites in mice [6,7]. Purified ARSeNL protein was pre-equilibrated with or without ATP and mixed with coelenterazine-h substrate just before imaging. The protein was enclosed in a custom plastic tube and placed subcutaneously in the left and right hindlimb areas, respectively, of a whole mouse (Figure 4) [40]. Even without shaving the animal, we measured a greater than 3-fold difference in the ratio, showing that the ATP-dependent signal was well-preserved when imaging through the tissue and fur (Figure 4). Thus, we established that ARSeNL is inherently excellent for filter-based ratiometric imaging and could be used for whole-animal imaging applications in principle.

**Figure 5:**
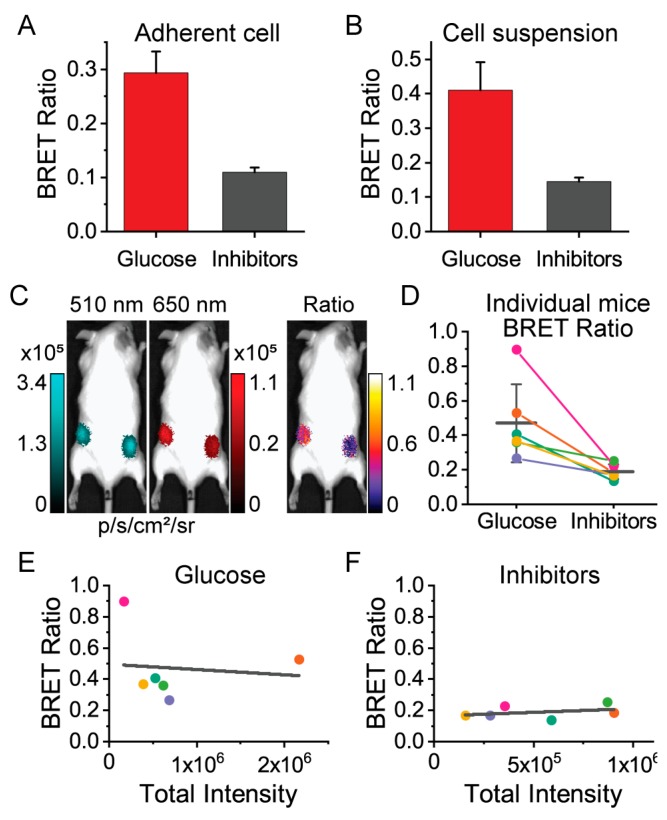
The ARSeNL BRET ratio reports differences in energy metabolism in live cells when imaged through tissue and fur and does not depend on sensor expression levels. The mScarlet/NanoLuc BRET ratio reports differences in ATP levels in (**A**) adherent HEK293A cells treated with glucose or an inhibitor cocktail of 2DG and oligomycin A (mean ± sd, *n* = 6, *p* < 0.001) or (**B**) live-cell suspensions treated with glucose or inhibitors (mean ± sd, *n* = 5, *p* < 0.001). (**C**–**F**) Whole-animal imaging of unshaved adult FVB mice injected with cell suspensions (*n* = 6). (**D**) Colored circles and lines connect glucose- and inhibitor-treated cells for individual mice. The black horizontal line and error bars are the populations mean ± sd (*p* = 0.013). There is no correlation between BRET ratio and total luminescence intensity (expression level) in the presence of glucose (**E**) R^2^ = 0.01 or inhibitors (**F**) R^2^ = 0.12.

We then moved to demonstrate that ARSeNL maintains its ability to detect metabolic differences in live cells in the context of whole-animal imaging. We first validated that we could measure differences in energy metabolism using adherent cell cultures. HEK293A cells expressing ARSeNL were seeded at different densities, from 62,500 cells/cm^2^ down to 4000 cell/cm^2^, to determine the sensitivity of detection in the SI Ami HT system. As expected, when imaging live cells, we found that the addition of glucose resulted in a ~3-fold difference in the ARSeNL ratio signal (0.29 ± 0.04, *n* = 6) compared to treatment with the metabolic inhibitors 2DG and oligomycin A (0.11 ± 0.01, *n* = 6) (Figure 5). Thus, the sensor effectively detects metabolic differences in live cells in a dish within the animal imager.

We next carried out ratiometric imaging of live cells injected in whole animals. Live cell suspensions of sensor-expressing HEK293A cells were obtained from independent cultures for each animal. Each cell suspension was split and treated with either glucose or metabolic inhibitors in parallel. Then, similar to previous studies [18,41,42], 10^6^ live cells were injected subcutaneously in the left and right hindlimb areas of adult FVB mice, respectively (Figure 5). Importantly, we measured the same ratio difference between glucose and inhibitor-treated cells that were observed for cell suspensions in a dish, even through the tissue and fur of unshaved mice (Figure 5). Of note, we used transiently transfected HEK293A cells to obtain a distribution of sensor expression levels across the independent trials. The total luminescence intensity provides an indicator of the total protein expression level, and critically we observed that the BRET ratio was dependent on the metabolic condition only and was independent of the level of luminescence intensity (Figure 5). Thus, we have demonstrated that ratiometric imaging of ARSeNL could be used to measure bioenergetic differences in live cells through tissue and fur in a manner that is independent of luminescence intensity decay and sensor expression levels.

## 4. Discussion

In this study, we constructed and screened a library of new luminescent bioluminescence resonance energy transfer (BRET) sensors, and we identified ARSeNL as a sensor using mScarlet and NanoLuc as a novel BRET pair that provides a much wider spectral difference between donor and acceptor peaks compared to previous sensors [43]. We showed that the BRET ratio is stable over time with different concentrations of protein and different numbers of cells, even when luminescence intensity decays due to substrate depletion, indicating that ratio measurements could reduce signal drift caused by variation in any of these factors in animals. Furthermore, we showed that the spectral properties of ARSeNL facilitate its detection in commercially-available filter-based whole-animal imaging systems that are commonly accessible, and we demonstrated that it reports metabolic differences in live cells when imaged through tissue and fur of whole adult mice. Furthermore, the conversion of FRET-based sensors to BRET-based sensors can be generally considered for improved quantitation in ratiometric whole-animal imaging. This approach will become even more effective in combination with the significant progress being made in the development of new orthogonal and red-shifted of luciferase-luciferin pairs [2,42], which will allow better measurement of physiology in animal models.

## Figures and Tables

**Table 1:** Library Screening Results.

Donor (Terminus)	Acceptor (Terminus)	Fold-Change in Ratio	
		FRET	BRET
NanoLuc (C)	mScarlet (N)	na ^‡^	5.12 ± 0.18
NanoLuc (C)	tdTomato (N)	na	4.83 ± 0.20
NanoLuc (C)	GRvT (N)	na	4.56 ± 0.29
NanoLuc (N)	mScarlet (C)	na	1.07 ± 0.21
NanoLuc (N)	tdTomato (C)	na	- *
NanoLuc (N)	GRvT (C)	na	- *
GeNL ^1^ (C)	mScarlet (N)	1.53 ± 0.01	1.90 ± 0.02
GeNL (C)	tdTomato (N)	- *	- *
GeNL (C)	RRvT (N)	1.39 ± 0.01	2.04 ± 0.01
CeNL ^2^ (C)	mScarlet (N)	1.88 ± 0.01	1.96 ± 0.01
CeNL (C)	tdTomato (N)	- *	- *
CeNL (C)	RRvT (N)	1.50 ± 0.01	2.07 ± 0.02
CeNL (C)	mNeonGreen (N)	1.86 ± 0.06	1.25 ± 0.02

^‡^ Not applicable to BRET-only sensors. * Poor or no expression. ^1^ GeNL is mNeonGreen-NanoLuc. ^2^ CeNL is mTurquoise2-NanoLuc. BRET: bioluminescence resonance energy transfer; FRET: Förster-type resonance energy transfer.

## Supplementary Materials

The following are available online at https://www.mdpi.com/1424-8220/19/16/3502/s1, Figure S1: Protein sequences, Figure S2: FRET ATP dose-response curves, Figure S3: BRET ratio stability of a control construct, Figure S4: BRET ratio stability of ARSeNL, Supplementary Methods.
