# Supplementary material for: Ratiometric BRET Measurements of ATP with a Genetically-Encoded Luminescent Sensor
Source: Sensors (Basel). 2019 Aug 10;19(16):3502. doi: 10.3390/s19163502 (PMC6721044; doi:10.3390/s19163502)
Supplement: Supplementary file 1 [file sensors-19-03502-s001.pdf]

Supplementary Material

Ratiometric BRET Measurements of ATP with a Genetically-Encoded Luminescent Sensor

Se-Hong Min[1,2], Alexander R. French[1,2], Keelan J.Trull[1], Kiet Tat[1], S. Ashley Varney[1], Mathew Tantama[1,2,3,*]

[1]Department of Chemistry,
[2]Purdue Institute for Integrative Neuroscience,
Purdue University, 560 Oval Drive, Box 68, West Lafayette, IN 47907, United States

[3]Department of Chemistry, Science Center, Wellesley College, 106 Central Street, Wellesley, MA 02481

*E-mail: mt4@wellesley.edu

CONTENTS

Supplementary Methods

Figure S-1. Protein sequences for sensors screened in the library
Figure S-2. FRET ATP-dose response curves for BRET-FRET sensors
Figure S-3. BRET ratio stability over time for the GRvT-NanoLuc control construct
Figure S-4. BRET ratio stability of ARSeNL (mScarlet-ε-NanoLuc) over time and protein concentration

Supplementary Methods

Plasmid Construction.

All sensor constructs used in this study are illustrated in Supplementary Fig. S-1. The constructs were cloned into the XhoI/HindIII sites of pRSETB bacterial expression vector or the XbaI/EcoRI sites of GW1 mammalian expression vector. The *Bacillus subtilis* ε subunit cDNA was amplified from a pRSET-ATeam1.03. NanoLuc cDNAs were amplified from Addgene plasmid #83926. Modified cDNAs containing mNeonGreen were amplified from a plasmid pcDNA3-mNeonGreen provided as a gift from Richard Day (Indiana University). CeNL cDNA was amplified from a plasmid pcDNA-CeNL (Addgene plasmid # 85199) provided as a gift from Takeharu Nagai. Modified cDNAs for mScarlet were amplified from a plasmid pCytERm_mScarlet_N1 (Addgene plasmid # 85066) provided as a gift from Dorus Gadella. A modified cDNAs for GRvT and RRvT were amplified from plasmids pBad-HisB-GRvT (Addgene plasmid # 87363) and pBad-HisB-RRvT (Addgene plasmid # 87364) provided as a gift from Robert Campbell. All sensors were constructed using standard cloning procedures. The cDNAs were amplified by PCR using Q5 polymerase (NEB), and vectors were double digested with restriction enzymes (NEB). Gibson assembly using NEB HiFi kit was used to assemble the plasmids for transformation. Plasmids generated in this study are distributed via Addgene.

Library Screen and Characterization of Purified Sensor *In Vitro*.

The fluorescence and luminescence of purified sensor proteins were investigated in assay buffer (50 mM MOPS-KOH, 50 mM KCl, 0.5 mM MgCl2, and 0.05% Triton X-100, pH 7.3) in a 96-well plate using a BioTek Synergy H4 multi-mode microplate reader at ambient temperature. For FRET library screening, 420/50 and 485/20 nm excitation filters and 485/20, 528/20, and 620/15 nm emission filters were used. For BRET screening, 20 μM coelenterazine-h was used as a substrate of NanoLuc and 450/50, 485/20, 528/20, and 620/15 nm emission filters were used. For characterization of mScarlet-ε-NanoLuc, 20 μM coelenterazine-h was applied to a reaction solution containing the purified sensor protein and 0-10 mM ATP in assay buffer. Luminescence spectra were scanned from 400 to 650 nm. Emissions of NanoLuc and mScarlet were measured using 450/50 and 620/15 nm emission filters, respectively, and the BRET efficiency was obtained by calculating the emission ratio of mScarlet/NanoLuc. Luminescence was also measured using a Spectral Instruments Ami HT with 510/20 and 650/20 nm emission filters (binning 2x2, FOV 25 cm, f-stop 1.2). Purified mScarlet-ε-NanoLuc protein in assay buffer with 0-10 mM ATP was imaged with a 1 sec exposure time after the addition of 20 μM coelenterazine-h. For measurements at different concentrations (1-100 nM) of mScarlet-ε-NanoLuc protein, the purified protein was mixed with 10 mM ATP or 0 mM ATP and imaged after the addition of 20 μM coelenterazine-h with a 5 sec exposure time for a total 30 min. Regions of interest were drawn over each well for all filter sets, and the average radiance was determined.

HEK293A Cell Maintenance.

HEK293A cells were cultured in Dulbecco's modified Eagle's medium containing 4.5 g/L glucose, 2 mM glutamine, and supplemented with 10% cosmic calf serum (HyClone) and were maintained at 37˚C in a 5% CO2 humidified incubator. Cells were transfected with plasmids carrying mScarlet-ε-NanoLuc by the calcium phosphate method[1].

Live Single-Cell BRET microscopy

HEK293A cells seeded onto glass-bottomed 96 well plates were allowed to adhere for two days before being transfected with sensor DNA. Cells were imaged 36-48 hours later on an Olympus IX83 microscope with an Andor Xyla 4.2 sCMOS camera, a Lumencor LED light engine, and a Prior motorized stage controlled by Andor iQ3 software. NanoLuc bioluminescence and mScarlet BRET were observed using 470/24m ET (Chroma) and 632/60m ET (Chroma) filters, respectively. An Olympus UApo N 340 40x objective (1.35 NA) was used for capture with exposure time 30 s and 2x2 binning each channel.

After removing the plate from the incubator, cell growth media was exchanged for imaging media (15 mM HEPES, 120 mM NaCl, 3 mM KCl, 3 mM NaHCO3, 1.25 mM NaH2PO4, 10 mM glucose, 2 mM CaCl2, 1 mM MgCl2). Imaging regions were determined using mScarlet fluorescence and DIC. Cell media was then exchanged twice for imaging media lacking glucose. Following this, Nano-Glo substrate (Promega #N2011) was prepared according to the manufacturer's guidelines with 1x furimazine concentration and added to the well. Cells were imaged for a three point baseline ($\Delta t = 2$ m) before having either metabolic inhibitors (10 mM 2DG + 2.5 μM oligomycin A) or vehicle (10 mM glucose + DMSO) treatments mixed into the wells. Cells were imaged until luminescence decayed to background (20-30 minutes). Image backgrounds were subtracted (rolling ball radus 100 pixels) before calculating the ratio images of the luminescence

channels using ImageJ[2]. ROIs were used to calculate each cell's mean ratio from the ratio image. For Figure 3, binary masks were generated using channel luminescence to remove pixels in ratio image corresponding to background. This is because the presence of zero value pixels in the luminescence background creates non-real pixel values in the ratio image. After normalizing each cell to its own baseline, the average time course of 6-9 cells in an independently transfected well was used as a single rep. Four reps were acquired for the drug condition and three for the vehicle.

Bioluminescence Cell Imaging.
HEK293A cells were transfected with plasmids carrying mScarlet-ε-NanoLuc by calcium phosphate method[1]. One day after transfection, cells were passaged onto a 96-well plate at different cell densities from 1,250 to 20,000 cells per well. After an additional 24-48 hours, imaging was performed using a spectral Ami with 510/20 nm and 650/20 nm filters (5 sec exposure time, binning 2x2, FOV 25 cm, f-stop 1.2) immediately after the addition of 20 μM coelenterazine-h.

Bioluminescence Imaging of Mice.
For imaging of purified protein in mice, mScarlet-ε-NanoLuc protein pre-equilibrated with 0 mM or 10 mM ATP was mixed with 20 μM coelenterazine-h. The reaction mixture was enclosed in a custom plastic tube and placed subcutaneously in the left (0 mM ATP) and right (10 mM ATP) hindlimb areas of adult Balb/c mice carcass. The sequential imaging was immediately performed using a spectral Ami with 510/20 and 650/20 nm filters (30 sec exposure time, binning 2x2, FOV 25, f-stop 1.2). For imaging of live cells in mice, HEK293A cells expressing mScarlet-ε-NanoLuc were imaged 36-60 hours after transfection by calcium phosphate method. On the day of imaging, cells were dissociated by cell dissociation buffer (Gibco), pelleted, and resuspended in glucose-free DMEM at one million cells in 100 μL two days after transfection. The cell suspension was mixed with glucose and 10% CCS or metabolic inhibitors and incubated for 30 min at room temperature before imaged. Half of the manufacturer's suggested amount of Nano-Glo furimazine was added to the cell suspension mix and cells were imaged at a 96-well plate before they were injected into animal. The cell suspension mixture was injected subcutaneously into the fore- or hind limb areas of unshaved adult FVB mice carcass. Prior to imaging of the mice, additional half of the manufacturer's suggested amount of Nano-Glo furimazine was added. Mice were imaged immediately using a Spectral Ami at room temperature. Sequential imaging was performed using 510/20 and 650/20 nm filters (60 sec exposure time, binning 2x2, FOV 25, f-stop 1.2).

Data Analysis.
Screening data acquired on the Synergy plate reader was acquired using Gen5 and analyzed using Microsoft Excel. Live cell microscopy data was acquired using Andor iQ3 software and processed in ImageJ. Spectral Ami images were acquired from Aura imaging software (Spectral Instruments Imaging) and analyzed using ImageJ. A threshold mask was applied to eliminate background pixels. Pixel values obtained from ImageJ were converted to radiance (photons/sec/cm^2/sr) by multiplying conversion factor calculated from Aura software. All measurement data were analyzed and visualized using OriginPro 2018b, MATLAB R2017a, and/or Inkscape 0.91. All measurements are shown as mean ± sd. Unpaired, two-tailed Student's *t*-test was used for comparison between the means of two groups.

mScarlet-ε-NanoLuc	MVSKGEAVIKEF...GRHSTG-RHID-MKTVK...VAGK-ANEFM-LEDFV...CERILA	(ARSeNL)
tdTomato-ε-NanoLuc	MVSKGEEVIKEF...GRHHLF-RHID-MKTVK...VAGK-ANEFM-LEDFV...CERILA	
GRvT-ε-NanoLuc	MVSKGEEVIKEF...GRHHLF-RHID-MKTVK...VAGK-ANEFM-LEDFV...CERILA	
NanoLuc-ε-mScarlet	MVFTLEDFV...CERILA-RHID-MKTVK...VAGK-ANEFM-AVIKEF...GRHSTGGMDELYK	
NanoLuc-ε-tdTomato	MVFTLEDFV...CERILA-RHID-MKTVK...VAGK-ANEFM-EVIKEF...GRHHLFLYGMDELYK	
NanoLuc-ε-GRvT	MVFTLEDFV...CERILA-RHID-MKTVK...VAGK-ANEFM-EVIKEF...GRHHLFLYGMDELYK	
mScarlet-ε-GeNL	MVSKGEAVIKEF...GRHSTG-RHID-MKTVK...VAGK-ANEFM-VSKGEED...CERILA	
tdTomato-ε-GeNL	MVSKGEEVIKEF...GRHHLF-RHID-MKTVK...VAGK-ANEFM-VSKGEED...CERILA	
RRvT-ε-GeNL	MVSKGEEVIKEF...GRHHLF-RHID-MKTVK...VAGK-ANEFM-VSKGEED...CERILA	
mScarlet-ε-CeNL	MVSKGEAVIKEF...GRHSTG-RHID-MKTVK...VAGK-ANEFM-VSKGEEL...CERILA	
tdTomato-ε-CeNL	MVSKGEEVIKEF...GRHHLF-RHID-MKTVK...VAGK-ANEFM-VSKGEEL...CERILA	
RRvT-ε-CeNL	MVSKGEEVIKEF...GRHHLF-RHID-MKTVK...VAGK-ANEFM-VSKGEEL...CERILA	
mNeonGreen-ε-CeNL	MVSKGEEDNMA...WQKAFT-RHID-MKTVK...VAGK-ANEFM-VSKGEEL...CERILA	

Figure S-1. Protein sequences for sensors screened in the library. Sensors are listed in the same order as Table 1 of the main text. Abbreviated sequences are highlighted in red for RFPs, grey for the *B. subtilis* epsilon subunit, blue for NanoLuc, pale green for GeNL, teal for CeNL, and bright green for mNeonGreen. The short peptide linkers, RHID and ANEFM, flanking the epsilon subunit are not highlighted. In the construction of the sensors, short segments were deleted from the N- or C-terminus of the fluorescent protein when adjacent to a fusion site in order to remove putatively flexible regions.

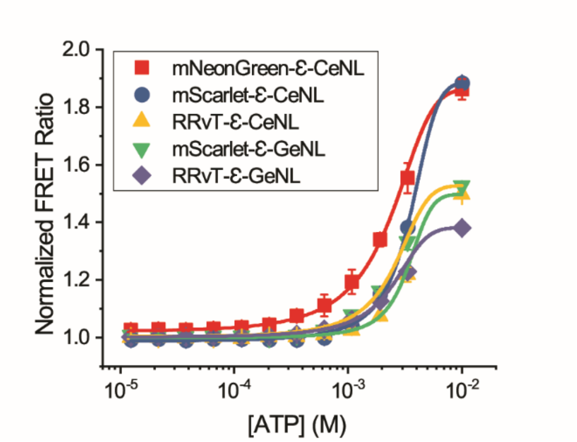

Figure S-2. FRET ATP-dose response curves for BRET-FRET sensors. ATP dose-response data from purified BRET-FRET sensor proteins. FRET ratio was calculated by emission ratio of acceptor fluorescence protein donor fluorescence protein from each sensor protein. Plots were fitted using a Hill equation. All BRET-FRET sensors have ~1.0 μM Kd value which is consistent with previous studies. Red square: mNeonGreen-ε-CeNL (NC), blue circle: mScarlet-ε-CeNL (SC), yellow triangle: RRvT-ε-CeNL (RC), green reverse triangle: mScarlet-ε-GeNL (SG), and purple diamond: RRvT-ε-GeNL (RG). mean ± sd, n=2-3, K_D is 1.0 ± 0.4 μM (NC), 1.0 ± 0.01 μM (SC), 1.0 ± 0.3 μM (RC), 1.0 ± 0.2 μM (SG), 1.0 ± 0.1 μM (RG).

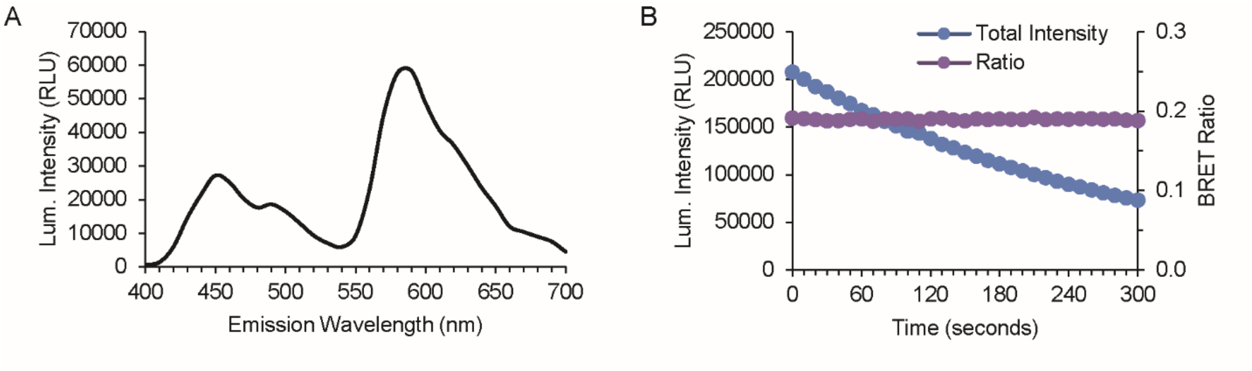

Figure S-3. BRET ratio stability over time for the GRvT-NanoLuc non-ATP binding control construct. (A) A control GRvT-NanoLuc construct lacking the ε subunit did not show improved BRET despite increased spectral overlap. (B) Similar to ATP-binding sensors reported in the main text, the GRvT-NanoLuc BRET ratio was stable despite the rapid luminescence intensity decay.

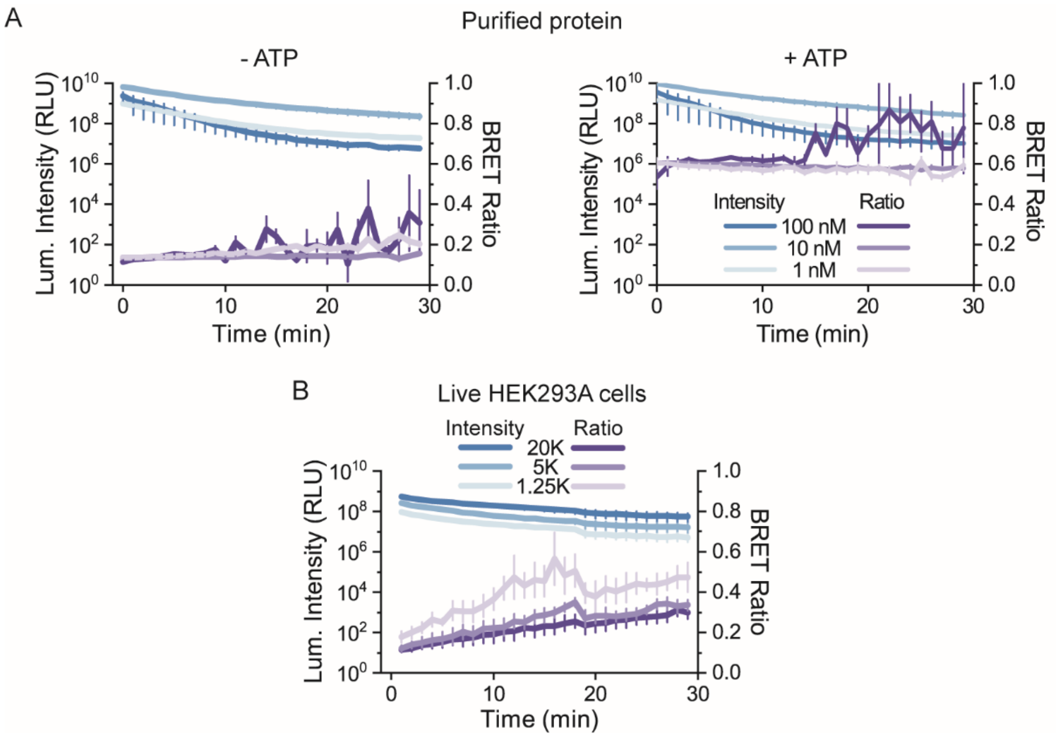

Figure S-4. BRET ratio stability of ARSeNL (mScarlet-ε-NanoLuc) over time and protein concentration. (A) Different concentrations (1-100 nM) of purified ARSeNL protein. BRET ratios from all concentrations were stable despite luminescence decay until 10 min after the addition of coelenterazine-h. The BRET ratio from 100 nM protein was noisy 10 min after the addition of the substrate because the higher enzyme concentration caused faster substrate consumption and faster luminescence decay so that background luminescence contributed to decreased signal-to-noise and increased variance in the BRET ratio when analyzing. However, overall, the BRET ratio is not directly affected by luminescence intensity. Error bars represent sd (n = 4). (B) Adherent HEK293A cells seeded at different densities from 20,000 cells to 1,250 cells per well in a 96-well plate. As cells were equilibrated after the addition of coelenterazine-h, the BRET ratio increased slowly for the first minutes, but it became stable despite ~10-fold changes in luminescence intensity. Error bars represent sd (n = 4).

References

(1) Jordan, M.; Schallhorn, A.; Wurm, F. M. Transfecting Mammalian Cells: Optimization of Critical Parameters Affecting Calcium-Phosphate Precipitate Formation. *Nucleic Acids Res.* **1996**, *24* (4), 596–601. https://doi.org/10.1002/jlcr.649.

(2) Schneider, C. A.; Rasband, W. S.; Eliceiri, K. W. NIH Image to ImageJ: 25 Years of Image Analysis. *Nat. Methods* **2012**, *9* (7), 671–675. https://doi.org/10.1038/nmeth.2089.
